# Active RAC1 Promotes Tumorigenic Phenotypes and Therapy Resistance in Solid Tumors

**DOI:** 10.3390/cancers12061541

**Published:** 2020-06-11

**Authors:** Pradip De, Brett James Rozeboom, Jennifer Carlson Aske, Nandini Dey

**Affiliations:** 1 Translational Oncology Laboratory, Avera Cancer Institute, Sioux Falls, SD 57105, USA; Pradip.De@avera.org (P.D.); Jennifer.Aske@avera.org (J.C.A.); 2 Departmental of Internal Medicine, SSOM, University of South Dakota, Sioux Falls, SD 57105, USA; 3 MSIV, SSOM, University of South Dakota, Vermillion, SD 57069, USA; Brett.Rozeboom@coyotes.usd.edu

**Keywords:** RAC1, solid tumors, resistance, CSC, EMT, proliferation, apoptosis, angiogenesis, metastasis, invasion

## Abstract

Acting as molecular switches, all three members of the Guanosine triphosphate (GTP)-ase-family, Ras-related C3 botulinum toxin substrate (RAC), Rho, and Cdc42 contribute to various processes of oncogenic transformations in several solid tumors. We have reviewed the distribution of patterns regarding the frequency of Ras-related C3 botulinum toxin substrate 1 (RAC1)-alteration(s) and their modes of actions in various cancers. The RAC1 hyperactivation/copy-number gain is one of the frequently observed features in various solid tumors. We argued that RAC1 plays a critical role in the progression of tumors and the development of resistance to various therapeutic modalities applied in the clinic. With this perspective, here we interrogated multiple functions of RAC1 in solid tumors pertaining to the progression of tumors and the development of resistance with a special emphasis on different tumor cell phenotypes, including the inhibition of apoptosis and increase in the proliferation, epithelial-to-mesenchymal transition (EMT), stemness, pro-angiogenic, and metastatic phenotypes. Our review focuses on the role of RAC1 in adult solid-tumors and summarizes the contextual mechanisms of RAC1 involvement in the development of resistance to cancer therapies.

## 1. Prologue

Ras-related C3 botulinum toxin substrate 1 (RAC1) overexpression has been implicated in multiple cancer cell phenotypes associated with tumor progression, metastasis, therapeutic resistance, and an overall worse prognosis for patients across a variety of solid tumors [1,2,3]. Despite years of development and innovation in the realm of cancer treatment, cancer cells have unique mechanisms to resist these advancements. RAC1 overexpression/overactivity is one proposed mechanism in therapeutic resistance. The RAC1 GTPase is a ubiquitously expressed member of the renowned Rho family of GTPases important across many cell-signaling processes relevant to cancer. As a GTP-ase, it serves as a molecular switch, cycling between the active, GTP-bound form and the inactive, GDP-bound form through the regulation of guanine nucleotide exchange factors (GEFs), GTPase-activating proteins (GAPs), and GDIs (guanine nucleotide dissociation inhibitors) [1]. Recently cancer-associated gain-of-function (GOF) mutations in RAC1 have been identified, which are responsible for aggressive tumor phenotypes and confer resistance to targeted therapies. In its active, RAC1-GTP form, it has demonstrated a remarkable contribution to diverse tumorigenic phenotypes. In this review, we focus on the role of RAC1 and its cell signaling networks in the promotion of five main tumorigenic phenotypes: anti-apoptotic, pro-proliferative, metastasis-associated/epithelial-to-mesenchymal transition (EMT), cancer stem cell (CSC), and pro-angiogenic (Table 1).

These phenotypes are by no means mutually exclusive and are merely an attempt to organize the existing literature regarding RAC1 in tumor progression and therapeutic resistance.

## 2. Anti-Apoptotic Signals of RAC1

The ability of cells to resist apoptosis is a hallmark of cancers [4]. We find that the predominant role of RAC1 in mediating the promotion of tumorigenesis, progression, and therapeutic resistance relates to its functions, promoting anti-apoptotic signaling pathways across solid tumors (Table 1). RAC1’s anti-apoptotic role has been observed in breast cancer, melanoma, non-small cell lung cancer (NSCLC), colorectal cancer (CRC), head and neck squamous cell cancer (HNSCC), and many others. Among these, RAC1 promotes tumor survival, progression, and therapeutic resistance through a network of anti-apoptotic cell-signaling pathways involving stress responses, cell membrane receptors, active RAC1 variants, reactive oxygen species (ROS) production, and B cell lymphoma/leukemia type 2 (Bcl-2) family activation.

### 2.1. Stress Response/Transcription Factors

A significant mechanism of anti-apoptosis is achieved through RAC1’s upregulation in response to cellular stress and the DNA damage response (DDR). Tumor cells under stress (radiation, chemotherapy, etc.) undergo the upregulation of RAC1, which activates a cascade of stress-responsive signals within the cytosol, cell membrane, mitochondria, and/or nucleus to promote the cell survival, proliferation, and migration. These may include nuclear factor kappa-light-chain-enhancer of activated B cells (NF-κB), stress-activated protein kinase (SAPK)/c-Jun N-terminal kinase (JNK), p38, signal transducer and activator of transcription 3 (STAT3), and Bcl-2 family mediated signals.

DDR, a complex cellular signaling algorithm involving cell cycle checkpoints, DNA repair, and apoptosis, is thought to be a cell’s response to genotoxic stress and is essential for the anti-apoptotic function of RAC1-overexpressed cancer cells. Importantly, RAC1 upregulation activates downstream NF-κB under cellular stress and is believed to be a notable way in which RAC1 serves its anti-apoptotic function in cancer cells. NF-κB itself has been widely implicated in cancer therapeutic resistance and progression, activating the expression of over 200 genes involved in pathways relating to cell proliferation, migration, and apoptosis/survival [5,6]. In a review of glioblastoma Rho-GTPase signaling, Fortin Ensign et al. discuss the role of RAC1 and its associated upstream GEFs in contributing to tumor progression and a pro-survival phenotype of glioblastoma cells via the downstream activation of the Bcl-2 family (through AKT2) and the RAC1 upregulation of downstream NF-κB [7].

In addition to its activation of NF-κB, RAC1 has been implicated in the resistance of cancer cells to radiation therapies through the associated downstream activation of ERK1/2 (extracellular-signal-regulated kinase), elevated expression of anti-apoptotic Bcl-2 family proteins, and decreased pro-apoptotic Bid and Bad proteins [6]. In a study of breast cancer cell populations exposed to hyper-fractionated radiation therapy, Hein et al. reported that RAC1 inhibition in a radiation therapy-resistant breast cancer population led to a decreased activity of ERK1/2 and NF-κB, decreased expression of anti-apoptotic Bcl-2 family proteins, and decreased expression of Poly (ADP-ribose) polymerase (PARP), suggesting the induction of apoptosis [6].

### 2.2. Bcl-2

Chong et al. recognized the relationship between RAC1 overexpression and ROS production, linking it to the anti-apoptotic Bcl-2 protein. The authors studied RAC1 overexpression in melanoma and lymphoma cells and suggested that it led to increased ROS and the phosphorylation of Bcl-2 (S70pBcl-2). This process subsequently permitted a direct, feedforward interaction and activation of RAC1-GTP between S70pBcl-2, sustaining anti-apoptotic signaling. Furthermore, they observed that the RAC1-GTP-S70pBcl-2 interaction could be inhibited via three main ways: (1) RAC1 inhibition, (2) BH3-mimetic inhibitors, and (3) the scavengers of ROS. They provide evidence for the apoptotic sensitivity of overexpressing tumors induced by the therapeutic targeting of RAC1 and/or S70pBcl-2 inhibition [8]. Hlavac et al. studied the anti-apoptotic and migratory effects of RAC1 and Bcl-2 family protein overexpression in glioblastoma cells. In their analysis, they demonstrate that the dual inhibition of RAC1 and Bcl-2 family proteins, Bcl-2 and Bcl-xL, led to a synergistic restoration of pro-apoptotic and anti-migratory behavior. Of note, they also observed that RAC1 inhibition (alone and with Bcl-2/Bcl-xL inhibition) led to the decreased viability of another anti-apoptotic Bcl-2 family protein, Mcl-1 (myeloid cell leukemia 1), providing another unique anti-apoptotic signaling mechanism attributed to RAC1 [9].

### 2.3. ROS

Another mechanism by which RAC1 overexpression contributes to therapy resistance is via its activation of Nicotinamide Adenine Dinucleotide Phosphate Hydrogen (NADPH) oxidase and the formation of ROS. In hepatocellular carcinoma (HCC), Zhou et al. describe that upregulated YES-associated protein (YAP) is involved in the signaling of RAC1. This Hippo-pathway effector protein led to the inhibition of cancer cell autophagy-related cell death through the intermediary signaling of the RAC1/ROS/ (mammalian target of rapamycin) mTOR pathway. More specifically, YAP both inhibited the pro-apoptotic RAC1-driven ROS production and sustained the pro-survival mTOR activity. Conversely, YAP’s inhibition, both in vitro and in vivo, sensitized HCC cell lines to chemotherapy by permitting autophagy signaling and eventual cell death through RAC1 activation/ROS production [10]. In ovarian and lung cancer cell lines, Wu et al. observed that RCC2 was able to inhibit the pro-apoptotic ROS production mediated by RAC1-hyperactivity in the context of the development of resistance to chemotherapeutic agents [11]. Furthermore, in tyrosine kinase inhibitor (TKI)-resistant NSCLC cell lines, Marcar et al. observed the interaction of RAC1 and PARP-1 on the production of ROS and cell survival. Interestingly, PARP-1 is reported to PARylate RAC1 and suppresses the RAC1/NADPH axis in the production of ROS, an action independent of the direct single-strand-break DNA repair mechanisms previously ascribed to PARP. Of note, the use of a combination of PARP inhibitor and TKI leads to the inhibition of the RAC1/NADPH/ROS axis and the sensitization of the TKI-resistant NSCLC cell populations to apoptosis [12]. Based on the seemingly conflicting data regarding RAC1 ROS production in either promoting or hindering apoptosis, it has been suggested that the degree of ROS production determines the tipping point in favor of survival or apoptosis.

Two stress-response kinases, p38 and JNK, have been recognized for their downstream role in RAC1 response to genotoxic stress, subsequent DDR, and resistance to cancer therapies [13,14]. In HER2-enriched breast cancer cells, Dokmanovic et al. suggest a role for RAC1 and JNK in resistance to first-line trastuzumab therapy. The RAC1 activity is increased in trastuzumab-resistant cell populations, and its inhibition leads to downregulated RAC1 and JNK activity in trastuzumab-resistant cell lines. Furthermore, they hypothesize that RAC1 mechanistically leads to trastuzumab resistance through three potential mechanisms: (1) directly interacting with the HER2 receptor on the inner side of the cell membrane, providing stabilization of RAC1-GTP and feedforward activation of the MAPK pathway; (2) activating downstream JNK; and (3) impairing the HER2 receptor endocytic downregulation by involvement with actin fibers of the cell cytoskeleton [14]. Alternatively, Su et al. suggest a radio-sensitization role of upregulated RAC1 and JNK cell signaling in response to radiation and a chemical compound, RP-4, that upregulates RAC1 in nasopharyngeal carcinoma cell lines. Their study showed that RP-4 upregulated RAC1 and NADPH, producing reactive oxygen species (ROS) and stimulating JNK/AP1 signaling for the promotion of apoptosis [15].

Interestingly, Li et al. suggest another DDR mechanism in which breast cancer cells overcome chemotherapeutic resistance via metabolism, stating the RAC1 overexpression after chemotherapy exposure led breast cancer cells to activate aldolase and ERK signaling, upregulating glycolysis and the pentose-phosphate pathway. The authors propose that resistant cancer cells use this process to alter their metabolism toward nucleotide renewal and availability under the stress of DNA damage caused by chemotherapeutics [16]. Although the exact mechanism of the RAC1-mediated development of resistance is not clear, the role of RAC1 will be interesting to study considering the relationship of tumor cell metabolism and the production of ROS following radiation, DNA-damaging agents, and various chemotherapeutic agents.

The spatio-temporal effects of RAC1 are especially important in cancer tissue’s response to cellular stress, specific DNA damage, and the DDR. Sandrock et al. demonstrated that activated RAC1-GTP could relocalize from the cell membrane to function within the nucleus and provided the transporter, KPNA2, mediating it [17]. RAC1 deficiency in hepatocytes is reported to play a protective anti-apoptotic role acutely after topoisomerase II inhibitors’ exposure alongside the harmful inflammatory response that occurs subacutely [18]. Skvortsov et al. built upon this work by describing the RAC1 expression and localization with respect to the stage of the HNSCC samples. They observed an increased RAC1 overexpression and nuclear RAC1 localization in cancerous HNSCC tissue in contrast to normal tissue. Furthermore, radiation exposure upregulated both the cytoplasmic and nuclear RAC1 expression, with the most dramatic increase in expression within resistant HNSCC cell populations. This RAC1 overexpression was accompanied by the increased clonogenic survival of the HNSCC cells as well [19].

### 2.4. Receptor Tyrosine Kinase (RTK) Pathways

As mentioned previously, RAC1 is frequently activated by upstream effectors. The RTKs, G-protein-coupled receptors (GPCRs), and other growth factor receptors are major players in this process. Specifically, the activation of upstream (phosphoinositide 3-kinase) PI3K and MAPK pathways are important to mention. Bright et al. suggest a necessary and complementary role for RAC1 and MAPK signaling in soft tissue sarcoma cell populations’ resistance to apoptosis under the frequent endoplasmic reticulum stress that occurs during oncogenic cellular proliferation. They report the individual and combined knockdown of mutated constitutively active forms of RAC1-P29I and NRAS-Q61 sensitizing the cell lines to ER stress.

Furthermore, MAPK activation was believed to be the converging mediator of co-dependent RAC1 and NRAS overactivity [20]. Zeng et al. studied the function of RAC1 in esophageal squamous cell carcinoma (ESCC) in promoting resistance to platinum chemotherapy. They observed that RAC1 inhibition led to the decreased expression of several glycolytic enzymes which are present in aerobic glycolysis and are believed to promote the resistance of cancer cells to chemotherapies. Based on their compiled data, the authors suggest that RAC1 regulates AKT1 (either directly or indirectly via mTOR1/2 binding), which serves as the primary regulator of aerobic glycolysis and therapy resistance [21]. As mentioned previously, another AKT isoform, AKT2, plays an anti-apoptotic role downstream of RAC1 by binding to the Bcl-2 family of pro-survival proteins [7]. Interestingly, this may be a link to the downstream anti-apoptotic signaling believed to occur in melanoma cell populations harboring mutant RAC1^P29S^ [22]. Zang et al. provide further evidence in prostate cancer cells for the role of RAC1 overexpression as an activator of the AKT/mTOR pathway. In their study, the RAC1 gene knockdown inhibited the AKT phosphorylation by upstream inputs of (Speckle-type POZ (pox virus and zinc finger protein) protein) SPOP-mutant therapy-resistant prostate cancer cell lines [23].

### 2.5. RAC1b Variant

Another contribution of RAC1 to the oncogenic transformation of cancer cells occurs through the RAC1b splice variant. The constitutively active, GTP-bound variant of RAC1, RAC1b, is preferentially expressed in both colon and breast cancers, enhancing the downstream activation of NF-κB and the induction of cyclin D1 expression in a variety of solid tumors [24]. Recently, Goka et al. described RAC1b overexpression in colorectal cancer and its role in resistance to tumor progression and resistance to current standard-of-care chemotherapy regimens for the treatment of advanced colorectal cancers. They demonstrated that colorectal cancer cells could develop resistance to chemotherapy via a stress-like response to DNA damage. In response to oxaliplatin and 5-FU, colorectal cancer cells can overexpress RAC1b, which activates downstream NF-κB expression, thereby promoting cell survival gene transcription. During the validation of their hypothesis, the authors observed that RAC1b-knockdown mice were sensitized to oxaliplatin and 5-FU treatment [24]. Interestingly, however, RAC1b alone is insufficient to promote tumor initiation in the lung. It can synergize with (Kirsten rat sarcoma viral oncogene homolog) KRAS for promoting tumorigenesis in NSCLC [25]. Although the role of RAC1 in the resistance setting is clearly emerging, almost all the reports are limited to preclinical studies.

## 3. Pro-Proliferative Signals of RAC1

RAC1 overexpression has been implicated in cancer cell proliferation (a hallmark of cancer), progression, and resistance. To achieve this function in solid tumors, RAC1 upregulates mitogenic growth factor signaling, stress response signaling, and the regulation of cell cycle checkpoints. Pro-proliferative effects are realized through a variety of stress-related cell-signaling proteins, splice variants [6,19,24], and the mitogenic/oncogenic PI3K and MAPK cell signaling pathways [14,26,27]. The pro-proliferative function of activated RAC1 by virtue of which it contributes to cancer progression and therapy resistance involves stress response proteins, including NF-κB, JNK, and p38. RAC1 can upregulate the downstream activity of the widely recognized transcription factor, NF-κB. Additionally, RAC1 contributes to the regulation of the cell cycle checkpoints G_1_/S and G_2_/M and cyclin D1 expression in the promotion of cellular proliferation. In NSCLC cell lines, Gastonguay et al. specifically investigated its pro-proliferative effects using RAC1-silencing RNA and a RAC1 inhibitor, NSC23766. In their analysis, they observed that RAC1 inhibition led to the arrest of cells in the G_1_ phase and significantly reduced colony proliferation in both anchorage-dependent and anchorage-independent ways. Importantly, it failed to demonstrate the regulation of the G_2_/S checkpoint proposed by others, yet cited multiple methodological reasons for this observation [5]. In another NSCLC study involving gefitinib-resistant cell populations, Zhang et al. observed the increased expression of RAC1, miR-135a, and phosphorylated members of the PI3K pathway. RAC1 was positively regulated by miR-135a and likely served an intermediary role between miR-135a and the activation of the PI3K pathway in promoting resistance [28]. However, the exact mechanism of the cellular signal remains unknown.

### 3.1. PI3K

Among the mitogenic signaling pathways linked to tumorigenesis, progression, and resistance, PI3K and MAPK are two fundamental targets in solid tumors. In glioblastoma cell populations that frequently harbor epidermal growth factor receptor (EGFR) mutations and upregulation, Karpel-Massler et al. studied the oncogenic EGFR pathway and RAC1’s role in therapeutic resistance to EGFR TKIs such as erlotinib. In their publication, they provide evidence for a synergistic anti-proliferative effect of combined EGFR and RAC1 inhibition and suggest that this occurs via the downregulation of the EGFR downstream PI3K and MAPK pathways [29].

### 3.2. MAPK

Like the PI3K pathway, the MAPK pathway has also been recognized in promoting the proliferation and tumor progression of solid tumors. As mentioned in the anti-apoptosis section, Dokmanovic et al. demonstrated that mutant RAC1^G12V^ reduced the sensitivity of HER2+ breast cancer cells to trastuzumab and suggested a role of RAC1 in overcoming trastuzumab therapy in breast cancer cell populations via RAC1’s binding to the intracellular domain of the HER2 receptor, its stabilized activation, and the subsequent activation of the MAPK pathway [14]. Wang et al. provide another potential mechanism in which RAC1 contributes to HER2+ breast cancer therapy resistance via a mutant HER2/RAC1/JNK/AP1/TGF-β (Transforming growth factor beta) pathway. They emphasize the causal role of the overexpressed components of this pathway to upregulated autocrine and paracrine TGF-β production in promoting tumor progression [26]. Hou et al. provide further support of RAC1’s contribution to oncogenic HER family signaling in cellular proliferation. In their analysis, the authors implicate RAC1 in the context of the phosphorylation of EGFR/HER1 and the upregulation of its downstream mitogenic signaling upon exposure to high glucose levels [27].

As mentioned previously, RAC1 overexpression can be a downstream result of upstream regulators such as GEFs, RTKs, or other effectors. In breast cancer cell lines, Cho et al. show evidence of overexpressed MST3, a protein kinase linked to cell growth, apoptosis, and migration, promoting cell cycle progression and growth through an (Mammalian STE20-like protein kinase 3) MST3/VAV2/RAC1/CyclinD1 pathway. Conversely, the introduction of a RAC1 inhibitor to the axis suppressed the proliferative tendency of these cell lines [30]. In multiple solid tumor cancer cell lines, Yue et al. report a mutant gain-of-function p53 (mutant p53 GOF) as another upstream activator of RAC1 and a pro-proliferative mechanism in tumorigenesis. The genetic knockdown or double negative expression of RAC1 in these cells abrogated the tumorigenic effects of mutant p53 GOF in the cancer tissue [31]. Consistent with these results, Yue et al. demonstrated a mutant p53 GOFs’ pro-tumorigenic phenotype through a p53-GOF/RAC1/AKT axis. Upon the RAC1 inhibition of mutant p53 GOF cell lines, the downstream AKT phosphorylation and the mutant p53 GOF tumor-promoting phenotypes were inhibited [32].

### 3.3. RAC1b

The overexpression of RAC1b in CRC cells promoted proliferation by the induction of cyclin D1 expression [24]. Goka et al. demonstrated that RAC1b inhibition with and without oxaliplatin therapy led to the decreased in vitro and in vivo growth of their CRC lines.

## 4. Gain-of-Function Mutation of RAC1

**Figure 1:**
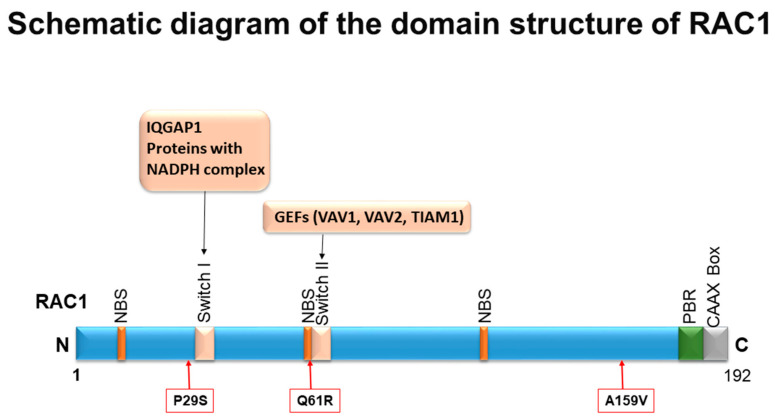
Schematic diagram of the domain structure of RAC1: Different domains of RAC1 include Nucleotide Binding Sites (NBS), switch I, switch II, Poly-Basic Region (PBR), and the CAAX box. The most important missense mutations in the context of oncogenic transformation are presented in the box, and the positions are indicated with arrows. Switch I primarily interacts with downstream effectors of RAC1, e.g., IQGAP1 and proteins in the NADPH complex. Switch II interacts with the RAC1-activating protein, guanine nucleotide exchange factors (GEFs). Switch II is the site where RAC1 becomes activated in its GTP-bound state. The diagram shows where effectors bind and where GEF proteins bind in switch I and II domains.

Though RAC1 amplification predominates within most solid tumor types, the overexpression of the active RAC1 hotspot mutant, RAC1^P29S^, has been implicated in metastatic cutaneous melanoma as a prominent mechanism of resistance, cell proliferation, and migration [22]. From a pharmacological standpoint, Watson et al. demonstrated RAC1^P29S^ to contribute resistance to previously targeted BRAF inhibitor therapy in mutant-BRAF cell lines. The authors validated this pro-survival phenotype by demonstrating the enhanced cell susceptibility to BRAF inhibitors and decreased phosphorylated MEK and ERK upon the silencing of the hotspot RAC1^P29S^. Interestingly, they observed RAC1^P29S^-expressing cell lines to possess enhanced resistance to both BRAF and MEK inhibitor therapy in vitro [33]. Recently, Lionarons et al. supported the ability of RAC1 to signal a pro-survival phenotype within cutaneous melanoma. In their analysis, they focus on an EMT phenotype (discussed later), which demonstrates overlap with the pro-survival phenotype, stating that RAC1 overexpression leads to both the downstream activations of an EMT transcription factor complex and the potential induction of an anti-apoptotic mechanism via the Bcl-2 family. A recent study demonstrated that melanoma cells carrying the RAC1^P29S^ mutation exhibit an augmented engagement of PAK1 (an immediate downstream effector of RAC1) in the presence of the RAC1^P29S^ mutant and are thus highly sensitive to the PAK1 inhibitor [34]. Other less prominent oncogenic RAC1 GOF mutations, RAC1^Q61R^ and RAC1^A159V^ (Figure 1), are reported in prostate and Head & Neck cancers, respectively [35].

It has also been reported that the active form of RAC1-GTP in prostate cancer cells promotes both survival and androgen receptor-independent cell growth [36]. Moreover, HNSCC tumors are associated with a higher rate of TP53 loss-of-function mutations and a lower rate of HPV infection, and patients with RAC1 alterations present more advanced clinical staging (T3/4 vs. T1/2) [37]. This evidence indicates that NHSCC tumors with RAC1 GOF mutations are more aggressive and a potential biomarker for cetuximab (FDA approved treatment option) resistance.

### ROS

As discussed previously, RAC1’s role in ROS production can have a seemingly dualistic role in the balance between cellular survival versus apoptosis; however, Ogrunc et al. propose another perspective on ROS production in pancreatic cancer cell lines, instead of focusing on proliferation versus senescence. The study provided evidence for a model in which the upstream oncogenic KRAS^G12D^-induced the activation of the RAC1/NOX4/ROS-axis or the NRF2-ROS axis promotes initial mitogenic signaling [38,39].

## 5. Metastasis-Associated/EMT

The transition of cancer cells from a differentiated (i.e., epithelial) phenotype to a less-differentiated mesenchymal phenotype is a hallmark of cancer progression. It is widely recognized as an important factor for invasive and migratory behavior during the metastasis of solid tumors. Currently, EMT is believed to be associated with overexpressed RAC1 [1,40,41]. The EMT phenotype is characterized by cells’ morphological transition from a differentiated (E-cadherin mediated cell-to-cell adhesion) state to a less-differentiated (mesenchymal N-cadherin, vimentin, laminin, lamellipodia formation, membrane ruffling, and decreased polarity) state via the reorganization of actin cytoskeleton networks [42]. RAC1 utilizes several pathways to achieve EMT, including upstream effector overexpression/overactivity, RAC1 overexpression, splice variants, upregulation of the wnt/β-catenin pathway, STAT3, ROS, stress response proteins, and phagocytosis.

### 5.1. Upstream Modulators

Upregulated upstream effectors of RAC1 are a frequent mechanism in which cancer cells obtain an invasive, migratory phenotype. In ovarian cancers, Hudson et al. describe overexpressed lipids, chemokines, metalloproteinases, growth factors, and other receptor ligands in the peritoneal fluid that stimulate both these receptors and downstream RAC1, leading to the metastatic phenotype [40]. Furthermore, alterations in the upstream receptors, GEFs, or GAPs can be influential as well. In cervical cancer cell lines, Wang et al. report the overexpression of a RAC1 GAP, SH3BP1, and its role in both the co-overexpression of RAC1/WAVE2 signaling, a known mechanism of invasion and migration, and chemotherapy resistance [43]. In a VAV1/KRAS^G12D^ pancreatic ductal adenocarcinoma cell line, Salaymeh et al. demonstrated that VAV1 is a potent activator of RAC1 overexpression, phenotypic metaplasia, and increased malignant potential [44]. In triple-negative breast cancer cells, we observed the ability of integrin (a transmembrane receptor, which facilitates the cellular adhesion of the actin cytoskeleton to the extra-cellular matrix) to promote a morphologically active, migratory phenotype via the RAC1-wnt/β-catenin pathway [45]. Yoon et al. reported in gastric adenocarcinoma cells the potential of the PI3K pathway’s influence as an upstream regulator in a PI3K/AKT/RAC1/JNK axis, citing evidence through various inhibition assays that the upregulation of the axis promotes EMT, invasion, and chemotherapy resistance phenotypes [46].

### 5.2. STAT3, NF-κB

One mechanistic pathway RAC1 functions to promote EMT occurs via either the direct phosphorylation and activation of STAT3 by its activated RAC1-GTP form or the indirect stimulation of autocrine IL-6 production (with eventual STAT3 activation) by a constitutively active RAC1^V12^ mutant. Zhou et al. observed in CRC that RAC1-GTP inhibition led to the decreased phosphorylation of STAT3, decreased N-cadherin expression, and increased E-cadherin expression. Furthermore, the constitutive activation of STAT3 abrogated the suppressive effect of RAC1-GTP inhibition on the EMT of the CRC cells [47]. Regarding the indirect mechanism of RAC1 activation of STAT3, Faruqi et al. report that a constitutively active form of RAC1 activates IL-6 cytokine and receptor production (via NF-κB), a known activator of STAT3. Importantly, however, the constitutively active form of RAC1 is a mutant RAC1^V12^ that could be a unique mechanism of STAT3 activation separate from wild-type RAC1 overexpression [48]. Gastonguay et al. report in NSCLC another potential mechanism for NF-κB in promoting migratory behavior (as a downstream target of RAC1) and tumor progression. In NSCLC cell migration assays, they demonstrate a statistically significant reduction in migration by RAC1 inhibition, an effect that they believe is due in part to NF-κB via the transactivation of migratory genes [5]. In addition to NF-κB, other stress proteins, such as p38 and JNK, have been implicated as downstream effectors in RAC1-mediated pro-invasive, migratory signaling in cancer cells [7].

### 5.3. RAC1b

Of note, the RAC1 splice variant, RAC1b, has been implicated downstream in an MMP3-initiated cascade leading to an EMT phenotype. In breast cancer cell lines, Radisky et al. report the ability of MMP3 (and other MMPs) to activate RAC1b overexpression, which is subsequently crucial to ROS production and the transcription of the EMT transcription factor, Snail [49]. Interestingly, Fortin Ensign et al. mention the ability of the RAC1-dependent activation of AKT2 to promote increased MMP-9 and invasive behavior in a review of Rho GTPase tumorigenic signaling in glioma cells [7].

### 5.4. Invadopodia

The ability of RAC1 to upregulate cell migration through lamellipodia formation is well established. Yet, Revach et al. describe in melanoma cells the ability of RAC1 to regulate “invadopodia” formation and ECM degradation in the promotion of metastasis. Interestingly, wild-type RAC1 overexpression led to invadopodia formation, and its fast-cycling, hyperactive mutant—RAC1^P29S^—suppressed the invadopodia process [42].

### 5.5. Phagocytosis

Yamazaki et al. demonstrate in oral squamous cell carcinoma that RAC1 mediates the phagocytosis of neighboring apoptotic cells via the actin cytoskeleton lamellipodia formation, promoting tumor progression and therapeutic resistance. The authors note that MFG-E8 accumulates near apoptotic cells and activates RAC1 through an integrin αvβ5-dependent mechanism [50]. In a similar light, Liu et al. implicate RAC1 as a mediator of endocytosis for IDH1-mutated glioma cells. Through a Rictor/mTOR2/RAC1/WAVE2 axis, these IDH1-mutant glioma cells overcome metabolite depletion by endocytosing beneficial ECM material, promoting tumor resistance [51].

### 5.6. RAC1^P29S^

In addition to their other observations in BRAF-altered melanoma, Lionarons et al. documented the potency of mutant RAC1^P29S^ to activate a downstream transcriptional WAVE-ARP2/3-SRF/MRTF cascade, leading to de-differentiation, enhanced cancer cell survival, and therapeutic resistance [22].

### 5.7. RAC1 Influences Cancer Stem Cell Property

Existing literature purports a positive regulatory role for RAC1 in the CSC phenotype. However, other regulatory molecules such as microRNAs (miRNAs) are present in the cellular milieu, providing effects on this phenotype as well. Reports are conflicting as to whether miRNAs serve stimulatory and/or inhibitory functions in tumor progression. In the case of prostate CSCs, Liu et al. observed that the artificial overexpression of miRNA-141 in prostate CSCs served to negatively regulate the primary properties of CSCs in tumor regeneration, progression, invasion, and metastasis. Furthermore, it promoted the differentiated epithelial state over the mesenchymal state [52]. Likewise, in liver CSCs, Jiang et al. observed that miRNA-135 overexpression led to the suppression of the de-differentiation and renewal capabilities of the CSCs via the direct inhibition of RAC1. Upon simultaneous RAC1 and miRNA-135 overexpression, the CSC phenotype was renewed [53]. In colorectal cancer cells, Rao et al. demonstrate the tumor-suppressive activity of SEMA3F via the inhibition of RAC1-GTP activity and other pro-CSC protein factors. Upon the overexpression of SEMA3F in the same tissue, a reduction in LGR5+ CSCs was observed [54]. Likewise, Yoon et al. reported a similar pro-CSC role of RAC1 overexpression in gastric adenocarcinoma cells and CSCs, promoting chemotherapy resistance. By inhibiting RAC1 in these populations, the self-renewal transcription factor, Sox-2, and its associated CSC phenotype were suppressed [46]. Lastly, Akunuru et al. provide further evidence of the overexpression of RAC1 in the more aggressive tumor phenotypes and the beneficial, anti-tumorigenic effect of its inhibition in both general and CSC NSCLC populations [55]. Importantly, the authors also describe difficulty in characterizing CSCs for testing their hypotheses. Other literature suggests a potential for a dynamic spectrum between CSC and non-CSC extremes. The functional relationship between the CSC and RAC1 in solid tumors appear to involve the EMT phenotype. The CSC phenotype shows a partial overlap with the EMT phenotypes, and thus the aspects of the CSC discussed may reiterate previous concepts.

## 6. Pro-Angiogenic Signals of RAC1

**Figure 2:**
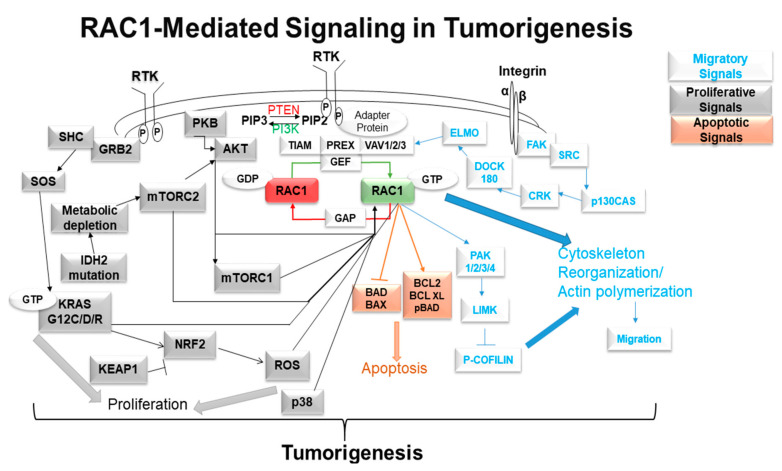
RAC1-mediated signaling in tumorigenesis: the cartoon presents the cellular signaling involving the activation of RAC1 in solid tumors. Following the activation of RAC1, a number of oncogenic cellular pathways are activated in tumor cells. The specific modes of involvement of the pathways and phenotypes are presented in the context of different mechanisms of activation(s) of RAC1 in different organ-type cancer(s).

Cancer-related angiogenesis is defined by the ability of tumor tissue to grow new blood vessels and obtain blood supply (neovascularization). It is a hallmark of cancer and is associated with a poor prognosis in patients with solid tumors. Several proteins have been cited as pro-angiogenic factors in the progression of cancer, including RAC1, MMPs, TIMP, and Nck1 [56]. As mentioned in the previous EMT section, MMPs degrade the ECM and, in this process, release pro-angiogenic factors such as VEGF, TGF-β, and others, signaling a downstream cascade involving RAC1. In ovarian cancer, Gonzalez-Villasana et al. demonstrate the ability of zoledronic acid to inhibit RAC1 activity and suppress a larger RAC1/PAK1/MMP-2 signaling pathway and ultimate pro-angiogenic phenotype [57]. Later, in cervical squamous cell cancer, Pei et al. implicated the signaling protein, Nck1, in the upstream activation of a Nck1/RAC1/PAK1/MMP-2 pro-angiogenic axis [58]. Lastly, Behelgardi et al. recently demonstrated further support for RAC1 as an important mediator in the pro-angiogenic VEGF cascade of highly metastatic breast cancer cell lines. The authors reported VEGF to activate both VEGFR1 and VEGFR2, signaling multiple different downstream effector pathways involved in invasion, migration, and angiogenesis, including the pro-angiogenic RAC1/PAK1 pathway [59]. The RAC1-PAK1 pathway has been identified as one of the key players in the development of resistance to anti-VEGF (bevacizumab) and anti-VEGFR (sunitinib) therapy. The inhibition of this pathway overcomes resistance to bevacizumab/sunitinib and decreases cancer stem cell functions in prostate cancers [60]. A schematic diagram of RAC1 signaling in the tumor cells is presented in Figure 2.

## 7. Conclusions

Like other members of the Rho-family of GTPase, RAC1 has a limited transcriptional role in a tumor cell. Understandably, none of the RAC1-mediated cell signaling relevant to the development of resistance in different cancers has been associated with a direct alteration of the genome. The mechanism(s) involved in the manifestation of the RAC1-initiated development of various resistances so far reported are mediated through the cytosolic-membranous cell signals or reported intra-nuclear activity. It is evident from the literature that numerous RAC1 inhibitors have been studied preclinically; however, their efficacy appears hindered by both a short half-life of RAC1 itself and the high IC_50_ values of current inhibitors. Activated RAC1 (amplification/overexpression/GOF mutation) contributes to tumor progression and the development of resistance in different organ types of solid tumors (Table 1). The predominant phenotype affected by RAC1 in bringing out different resistance in solid tumors is “anti-apoptotic” followed by “pro-proliferative” and “metastatic-associated/EMT.” Additionally, the resistance to chemotherapy has been reported most in connection to the RAC1 effect on the anti-apoptotic phenotype. Thus, there appears to be a potential opportunity for the research and development of RAC1-guided inhibitory therapies to manage the tumor progression and resistance in solid tumors. Moreover, it is worth mentioning that the RAC1^P29S^ mutant in melanoma exhibits a higher expression of PD-L1, a ligand of immune-check-point protein, PD-1, that plays a fundamental role in immune evasion [61]. Taken together, we argue that a pharmacological intervention with RAC1 inhibitor (or its downstream effectors) in combination with anti-PD-L1/PD-1 antibody may provide a novel therapeutic opportunity in BRAF inhibitor-resistant melanoma patients. Gene Set Enrichment Analyses (GSEA) of RAC1 mutated HNSCC patients also showed a significant aberration of genes related to the immune response as compared to WTRAC1 tumors, including the pro-tumorigenic inflammatory cytokine, IL6 [37].

## Figures and Tables

**Table 1:** Involvement of ras-related C3 botulinum toxin substrate 1 (RAC1) in solid tumors: a survey of the current literature. This table provides a bird’s eye view of the phenotypes regulated by RAC1 and its downstream signals in different solid tumors pertaining to the development of various resistances.

Phenotype	Signaling Pathway	Resistance	Solid Organ	PMID #
Anti-Apoptotic	RAC1-GTP-S70pBcl-2	Chemotherapy	Melanoma	31103719
	NF-κB		Colorectal	30926638
	RCC2-RAC1-ROS		Lung and Ovarian	29321004
	RAC1-AKT or RAC1-mTOR1/2-AKT		ESCC	31314174
	RAC1 Nuclear Localization		Unspecified	23907156
	RAC1-SAPK/JNK		Unspecified	26437439
	RAC1-Aldolase, ERK-PPP		Breast	32193458
	RAC1 Nuclear Localization	Chemo-Radiation	HNSCC	24786604
	JNK/AP1	Radiation	HNSCC	30463023
	RAC1-ERK1/2; RAC1-NF-κB		Breast	27181206
	RAC1-AKT	Targeted Therapy	Prostate	28805822
	SAPK/JNK; HER2-MAPK		HER2+ Breast	19509242
	PARP1-RAC1-ROS		Lung	31216465
	RAC1-PDL-1		Melanoma	26176707
	MAPK	ER Stress	Unspecified	29329780
	RAC1 Nuclear Localization	General	Unspecified	19961560
	VAV-RAC1-Autocrine IL-6-STAT3		Unspecified	11470914
	YAP-RAC1-ROS-mTOR		Hepatocellular	31337986
	RAC1-AKT; RAC1-NF-κB		Glioblastoma	24109588
	RAC1^P29S^-MEK-ERK		Melanoma	25056119
	RAC1-Usp9X-Mcl1, Bcl2		Glioblastoma	30859392
Pro-Proliferative	RAC1-PI3K-AKT	Targeted Therapy	NSCLC	29386087
	EGFR-RAC1		Glioblastoma	23832120
	HRAS^G12V^-RAC1-autocrine TGF-β		Unspecified	20383197
	RAC1-PAK-MEK-ERK		Melanoma	29059171
	Review Article	General	Breast	32314182
	MST3-VAV2-RAC1-CCND1		Breast	26910843
	RAC1-EGFR		Breast	28670141
	RAC1-NF-κB		NSCLC	22549160
	Mutant p53-RAC1-AKT		Unspecified	32275841
	Mutant p53-RAC1		Breast, Prostate, CRC	28947497
	RAS-RAC1-NOX4-ROS		Pancreatic	24583638
	RAC1b-KRAS		NSCLC	22430205
	Unspecified		Prostate and HNSCC	26619011
	cSrc-RAC1-alphaPKC		Prostate	20203103
Metastatic-Associated/EMT	SH3BP1	Chemotherapy	Cervical	28786507
	RAC1-Sox2		Gastric	28461325
	RAC1-Snail1, Vimentin, N-cadherin, Twist1	Radiation	NSCLC	27877226
	Review Article	General	Ovarian	30261690
	Wnt/β-catenin-TIAM1-VAV2-RAC1		TN Breast	27902969
	RAC1-STAT3		Colorectal	29884911
	MMP3-RAC1b-ROS		Breast	16001073
	Invadopodia		Melanoma	26873115
	MFGE8-RAC1-DKK1		Oral	32320683
	VAV1-RAC1		Pancreatic	32277014
	Mutated IDH1-mTOR2-Rictor-RAC1-WAVE2		Glioma	32224866
	RAC1^P29S^-PAK, AKT, (WAVE2-ARP2/3-SRF/MRTF)		Melanoma	31257073
Cancer Stem Cells	miRNA-135-RAC1	General	Hepatocellular	30182377
	SEMA3F-RAC1-Wnt/β-catenin		Colorectal	25529012
	RAC1 Overexpression		NSCLC	21347385
	miRNA-141-RAC1		Prostate	28112170
Pro-Angiogenic	VEGFR-Prex1-RAC1-ERK	Targeted Therapy	Prostate	26923603
	Nck1-Rac1-PAK1-MMP2	General	Cervical	30442385
	Rac1-PAK1-p38-MMP2		Ovarian	25595279
	VEGFR1-PI3K-AKT-RAC1; VEGFR2-RAC1-PAK1		Breast	32072404
Phenotypes	Review Article	General	Solid Tumors	29548483
				31027363
				10647931
